# Chemotherapy in a Breast Cancer Patient Heterozygous Carrier of Ornithine Transcarbamylase Deficiency

**DOI:** 10.7759/cureus.8301

**Published:** 2020-05-26

**Authors:** Magda Palka-Kotlowska, Luis Cabezón-Gutiérrez, Sara Custodio-Cabello, PIlar Quijada-Fraile, Silvia Chumillas-Calzada

**Affiliations:** 1 Clinical Oncology, Hospital Universitario de Torrejón, Madrid, ESP; 2 Medical Oncology, Hospital Universitario de Torrejón, Madrid, ESP; 3 Unidad Pediátrica De Enfermedades Raras, Metabólicas-Hereditarias Y Mitocondriales, Hospital Universitario 12 de Octubre, Madrid, ESP

**Keywords:** urea cycle deficiency, ornithine transcarbamylase deficiency, chemotherapy, breast cancer

## Abstract

Urea cycle disorders (UCDs) are an unusual genetic condition that may lead to hyperammonemia in catabolic situations such as surgery, infections or chemotherapy administration. Without specific treatment, it causes life-threatening encephalopathy. We present the case of a young woman, heterozygous carrier of ornithine transcarbamylase deficiency (OTCD) with breast cancer, who was treated with surgery, chemotherapy, radiotherapy and hormone therapy while following a protocol to minimize the risk of metabolic decompensation due to her condition.

## Introduction

Urea cell cycle is the metabolic pathway that metabolizes ammonium to urea in the liver in order to excrete it via the renal function. Urea cycle disorders (UCDs) are genetic diseases which include carbamyl phosphate synthetase I (CPSI) deficiency, ornithine transcarbamylase deficiency (OTCD), argininosuccinate synthetase (ASS) deficiency, also called citrullinemia type I, argininosuccinate lyase (ASL) deficiency, also known as argininosuccinic aciduria, N-acetyl glutamate synthetase (NAGS) deficiency and arginase deficiency [1]. Those disorders could result in life-threatening metabolic decompensation during early childhood because of hyperammonemia. If the patient survives, serious neurological sequelae may appear. Because of its low incidence, a high index of suspicion must be present if the screening was negative, taking into account that OTCD and most of UCD are not included in newborn screenings. It is diagnosed in 1 in 35,000 newborns and its mortality is 24% in cases of neonatal-onset [2]. UCDs are autosomal recessive disorders with the exception of OTCD, which is X linked. Only 10% of female carriers are symptomatic because of the lyonization process (X chromosome metilation to inactivate a deficient gene) but homozygous males can be severely affected at any age.

Breast cancer is the most common cancer in women. It is diagnosed in 25% of new female cancer patients. It is the leading cause of cancer-related death in women (15%), followed by lung cancer and colorectal cancer [3]. In early-stage cases, screening, surgery, chemotherapy, radiotherapy, and hormone therapy have decreased mortality dramatically over the last 30 years.

## Case presentation

We present the case of a 40-year-old female, a former smoker. She is an asymptomatic carrier of OTCD, diagnosed after her newborn presented with this disorder and passed away at the age of three. She leads a normal life and diet, with no protein restriction. In June 2018, she was diagnosed with localized infiltrating ductal carcinoma of the right breast, Nottingham grade 2, positive for hormonal receptors (80%), HER2 negative, ki-67 20%. After a multidisciplinary committee, she underwent breast-conserving surgery in July. Final staging was pT2pN1[sn]M0, and adjuvant chemotherapy, radiotherapy, and hormone therapy were proposed. Concerns about the risk of adjuvant chemotherapy infusion were raised because of OTCD. Ten years overall survival of such cases of breast cancer cases is 90% and chemotherapy provides approximately 15% of this benefit [4]. Colleagues from a reference center for the metabolic disease were invited to breast cancer multidisciplinary committee of this patient. Thus, a specific protocol was followed to prevent hyperammonemia during chemotherapy treatment:

Firstly, based on the guidelines available for diagnosis and management of UCD [1,5], diet intervention was indicated to prevent metabolic decompensation: since the previous night to chemotherapy dose and for the next 24 hours, she had increased calories intake with a reduction of food enriched proteins. She was advised to have fruits and vegetables, rice, pasta, bread and cereals, oils, butter, sugar or honey and was advised to avoid meat, fish, dairy products, eggs, legumes and nuts. Twenty-four to 48 hours after chemotherapy, the patient could include in her diet half of the content of high biological value proteins. On the third day after chemotherapy, she could consume a normal food intake. Secondly, during chemotherapy treatment, an infusion of dextrose 10% solution to deliver 3 mg/kg/min of glucose was administrated until oral intake was guaranteed. In blood tests taken before each chemotherapy dose, ammonia plasma levels and venous blood gases were measured altogether with blood count, hepatic and renal function and electrolyte panel. And thirdly, febrile neutropenia was prevented with five doses of filgrastim after each doxorubicin and cyclophosphamide dose, initiating at least after 24 h of chemotherapy administration.

She underwent chemotherapy between September and February 2019, received four cycles of adriamycin 60 mg/m2 + cyclophosphamide 600 mg/m2 every three weeks and 12 weekly cycles of paclitaxel 80 mg/m2 intravenously. She tolerated the cycles well, with nausea but without vomiting, with asthenia grade 1, alopecia grade 2, and peripheral neurotoxicity grade 1. Ammonia plasma levels and venous blood gases were within normal limits during all chemotherapy treatment and she did not present febrile neutropenia or infection. She followed the diet counseling and neither lost nor gained weight during the treatment. Then, she received breast radiotherapy for six weeks and now she is on tamoxifen 20 mg per day, aiming 10 years of hormone therapy.

## Discussion

To our knowledge, there are no published case reports about breast cancer treatment in OTCD patients. Literature is scarce, including mainly children or patients with acquired hyperammonemia emulating UCD.

UCD is not usually an area of expertise of breast cancer multidisciplinary committees; therefore, expert guests are essential in cases like this. Colleagues from the reference center in metabolic disease recommended a specific protocol based on guidelines of management of UCD for the patient during the adjuvant chemotherapy. Such protocol is not routinely used in oncologic patients receiving treatment: patients without UCD can follow a normal diet without protein load restrictions and ammonia levels are not checked in blood tests before each cycle. As for febrile neutropenia, in breast cancer adjuvant therapy, primary prophylaxis with granulocyte-colony stimulating factor (filgrastim) is recommended, but it is not extended to all chemotherapy regimens. Awareness of UCD in this patient was very important because, if she had have developed symptoms such as loss of appetite, vomiting or encephalopathy, differential diagnosis would include this genetic disorder, and specific treatment would be the key to avoid life-threatening complications of UCD.

Clinical manifestations of UCD

Clinical presentation of UCD usually occurs in newborns after 24-48 hours of life, when human milk or formula feeding provides a protein load. It can present with somnolence, low core temperature, poor feeding, vomiting, and coma. This might be confused with sepsis, but the absence of risk factors and a non-diagnostic infection evaluation should prompt a high grade of suspicion of metabolic disorder. Some UCDs are part of the newborn screening programs, but OTCD is not detected in the usual neonatal screening tests [6]. In female carriers, it can present as late-onset in situations that lead to catabolic status (surgery, peripartum, intense exercise, infection, drugs such as steroids or valproic acid, or chemotherapy administration) [7]. Symptoms in those cases are subtle and consist of loss of appetite, vomiting, lethargy, and encephalopathy [1].

Diagnosis of OTC deficit

Abnormal elevation of ammonia plasma concentration together with a normal anion gap and normal plasma glucose concentration is a strong indication of UCD. Also, the plasma concentration of citrulline helps to discriminate between proximal and distal urea cycle defects, as citrulline is the product of the proximal enzymes (carbamoylphosphate synthetase I, ornithine transcarbamylase and N-acetyl glutamate synthetase) and a substrate for the distal enzymes (argininosuccinic acid synthetase, argininosuccinic acid lyase, and arginase I). Plasma citrulline is low in neonatal-onset OTCD and presents with low or low-normal concentrations in late-onset disease. If citrulline is low, urine orotic acid is increased in OTCD [8]. Plasma aminoacids arginine and ornithine are not elevated in OTCD.

Next-generation sequencing is the most common diagnostic technique because of its sensitivity, but there are others such as DNA mutation testing, array comparative genomic hybridization or chromosome microarray analysis [9]. Liver biopsy and urinary excretion of orotic acid after allopurinol administration are used only in selected cases where other methods have had negative results.

Treatment and case reports of hyperammoniemia in the context of OTC deficiency

OTC deficiency may be treated effectively with a low protein diet out of acute situations. Drugs that are routinely used for long-term treatment of UCDs include nitrogen scavengers (sodium benzoate, sodium PBA or sodium phenylacetate, glycerol phenylbutyrate), L-arginine, and L-citrulline [6,7].

Published cases of patients with OTC deficiency treated with chemotherapy are few:

Peters et al. describe a pediatric case of acute lymphocytic leukemia with fatal outcome after administration of pegylated asparaginase [10]. In this case, urine orotic acid was 1123.9 mmol/mol Cr (RR 0.4-1.2). This is an unequivocal sign of OTC deficiency. Patient's DNA was not available for molecular DNA testing. Testing of the asymptomatic biological mother revealed a pathogenic mutation in the OTC gene, c.830 G>A; p. Arg277Gln, previously reported with late-onset OTC deficiency. The authors suggest that in cases of a high risk of hyperammonemia related to the disease itself or to the treatment, screening with ammonia levels should be done prior to treatment initiation.

A molecular panel, including urea cycle genes prior to chemotherapy could identify late-onset UCD and lead to modifications in treatment prior to induction. Several published cases show acquired hyperammonemia emulating UCD without a proven genetic alteration but induced by chemotherapy drugs. Several are associated with chemotherapy drugs and steroids in patients with hepatic and hematologic malignancies undergoing intensive cytoreductive treatment or bone marrow transplantation [11-19]. Causes are multifactorial, but catabolic state induced by chemotherapeutic agents that overwhelm the capacity of the urea cycle is possibly one of the main milestones.

Other cases describe acquired deficit of OTC because of activation of the competing enzyme ornithine decarboxylase (ODC) that deviates ammonia metabolism from synthesis of urea to synthesis of polyamines. The cytotoxic drugs most commonly associated with hyperammonemia are 5-fluorouracil, cyclophosphamide, oxaliplatin, vincristine, etoposide, anthracyclines, busulfan, methotrexate, topotecan, vinorelbine, gemcitabine, cytarabine, L-asparaginase, steroids, sunitinib, sorafenib, and regorafenib [20]. Some of them are frequently used in breast cancer treatment.

In our case, we were aware of the OTC deficit of our patient, so close collaboration with a reference center for the metabolic disease was important to prevent complications due to this condition during the treatment.

## Conclusions

The knowledge and degree of suspicion of a rare disease may prevent serious complications for oncologic patients. When dealing with this patient, bibliography and evidence were scarce. Our goal is to share medical knowledge and experience in rare cases as OTCD to be able to treat patients safely for whom we do not have clinical trials. A multidisciplinary committee and awareness of the symptoms, diagnosis and management of OTCD carriers of our patient were crucial to prevent life-threatening complications.
